# Data Analysis of Psychological Approaches to Soccer Research: Using LDA Topic Modeling

**DOI:** 10.3390/bs13100787

**Published:** 2023-09-22

**Authors:** Jea Woog Lee, Doug Hyun Han

**Affiliations:** 1 Intelligent Information Processing Lab, Chung-Ang University, Seoul 06974, Republic of Korea; yyizeuks@cau.ac.kr; 2 Department of Psychiatry, Chung Ang University Hospital, Seoul 06974, Republic of Korea

**Keywords:** soccer, psychology, research trends, data science, topic modeling, bibliometric

## Abstract

This study identifies the topical areas of research that have attempted a psychological approach to soccer research over the last 33 years (1990–2022) and explored the growth and stagnation of the topic as well as research contributions to soccer development. Data were obtained from 1863 papers from the Web of Science database. The data were collected through keyword text mining and data preprocessing to determine the keywords needed for analysis. Based on the keywords, latent Dirichlet allocation-based topic modeling analysis was performed to analyze the topic distribution of papers and explore research trends by topic area. The topic modeling process included four topic area and fifty topics. The “Coaching Essentials in Football” topic area had the highest frequency, but it was not statistically identified as a trend. However, coaching, including training, is expected to continue to be an important research topic, as it is a key requirement for success in the highly competitive elite football world. Interest in the research field of “Psychological Skills for Performance Development” has waned in recent years. This may be due to the predominance of other subject areas rather than a lack of interest. Various high-tech interventions and problem-solving attempts are being made in this field, providing opportunities for qualitative and quantitative expansion. “Motivation, cognition, and emotion” is a largely underrated subject area in soccer psychology. This could be because survey-based psychological evaluation attempts have decreased as the importance of rapid field application has been emphasized in recent soccer-related studies. However, measuring psychological factors contributes to the study of football psychology through a new methodology and theoretical background. Recognizing the important role of psychological factors in player performance and mental management, as well as presenting new research directions and approaches that can be directly applied to the field, will advance soccer psychology research.

## 1. Introduction

It is common knowledge that soccer is the most popular participation and spectator sport in the world today [1]. This means that there are many players, teams, and fierce competition. As with any sport, soccer teams strive to win in the face of fierce competition, and soccer is widely studied by researchers owing to its popularity. Many professional soccer clubs have emphasized the importance of sports psychology in improving their performance [2,3].

In recent years, the role of psychology in various subfields related to soccer has become very important [4]. Many efforts have been made to apply psychology to soccer, including administrative efforts such as the creation of the “Psychology for Soccer” strategy introduced by the English Football Association in 2011 [5]. These attempts have served as valuable milestones in understanding how psychological exploration can impact players’ performance and lives [6]. Research has also repeatedly shown that a healthy body and mind can influence players’ lives after their playing careers [7]. These findings are relevant, not only to professional soccer clubs, but also to a wide range of soccer workers, including youth players and referees [8,9]. As such, much of the research has been integrated into educational programs for coaches, players, and support staff to teach them psychological concepts and, most importantly, connect them with practicing football and sports science psychologists.

The above-mentioned reviews have all made valuable contributions to the current understanding of the research conducted in the field of sport and exercise psychology; therefore, there must be a large quantitative and qualitative accumulation of psychological research that has been continuously explored for the development of soccer over the years. Klarin (2019) reported that a macro view must be taken from a position of reflection and introspection for the discipline’s long-standing tradition to become more rooted and more forward-looking [10].

There are ways to explore the intellectual structure of research through, for example, literature review, content analysis, and meta-analysis, but a framework that integrates large disciplines such as psychology constitutes natural language processing [11]. The development of computing power, represented by the effective collection, storage, processing, and real-time sharing of data, due to the advent of the information age, has made such a framework possible [12].

In particular, the emergence of the concepts of artificial intelligence and big data has made it possible to derive new insights from vast and unstructured data through high-level analysis [13]. Natural language processing is one of the methods that can realize these expectations, and it is being used in various academic fields. In this study, we used the natural language processing techniques of text mining and topic modeling. Topic modeling is a technique that analyzes a dataset of documents and discovers latent topics through the derived unstructured text to explore key issues and controversies in a particular field [14,15].

Compared to other literature review methodologies, topic modeling is advantageous, owing to its ability to explore the published literature in a field in depth. [16] reported that the keyword-driven segmentation of topic areas can reveal the major theories, methodologies, and themes that shape the sport and exercise psychology literature and how these various aspects relate to each other. By building on this body of work, we could further solidify the findings of the literature and uncover meaningful insights into new areas of research.

The purpose of this study is to identify research topics in the field of soccer that have attempted to take a psychological approach to the study of soccer, and to identify their growth and stagnation, as well as their contributions and prospects for soccer development. First, this study set the scope of the collection database as the Science Citation Index Expanded, Social Sciences Citation Index, and Art & Humanities Citation Index indexed in the Web of Science (WOS) database. The first soccer study with a psychological approach found in the database was in 1990. Therefore, a database of articles collected over a 33-year period from 1990 to 2022 was established and topic keywords, topics, and topic areas were extracted. Second, we identified macro-level research trends in psychological approaches to soccer research by identifying key keywords, topics, and topic areas through topic modeling analysis. In addition, it contributed to providing insights into how the identified research topics played a role in different aspects of soccer fields. Third, we identified active and stagnant research areas through statistical analysis of changes in the frequency and share of each topic area. Then, we analyzed various backgrounds for this phenomenon and predicted future responses, changes, and research directions.

## 2. Materials and Methods

### 2.1. Data Collection

In this study, WOS was selected as the database to collect the articles. Cheng et al. (2018) reported that WOS is rich in data volume, including complete coverage of more than 10,000 journal citations, is well recognized in the academic community, and supports batch extraction of article information, which enables fast and accurate data processing [17]. In addition, the taxonomy of WOS and the indexes of other academic information databases have been criticized for their lack of ability to generate consensus measures for scientific measurement studies [18]. In deciding on WOS as a data source, we considered the arguments of various topic modeling-related prior studies. The keywords used in the search were “soccer” and “football”, and 53,671 results were retrieved by entering the search term “soccer or football” in the WOS database. Next, from the list of all soccer-related articles retrieved, 4692 articles in the field of psychology were extracted using WOS’s filtering capabilities. From these 4692 articles, a further 1844 articles of low relevance were excluded by hand monitoring of the collected articles by experts in soccer, sports psychiatry, and sports psychology, including the researcher. Next, we also excluded 237 studies that focused on rugby, American football, and Australian football. Finally, we excluded 748 articles that were not peer-reviewed (e.g., news articles, letters to the editor, research reports, conference presentations, and books), leaving 1863 articles for analysis.

### 2.2. Data Preprocessing

Natural language processing involves synthesizing a set of processes that intuitively derive meaning from unstructured words in text documents organized into sentences. Furthermore, the natural language processing process of academic research involves analyzing abstracts that summarize the key content of the study [19]. Therefore, in this study, the procedure of analyzing the collected article abstracts was performed. Morphological analysis, one of the natural language processing techniques, was performed using the original data of the collected article abstracts. Through morphological analysis, 17,665 words were extracted from 1863 article abstracts.

Most natural language processing analysis methods, including topic modeling, are preceded by data preprocessing techniques to identify only the words that will be used in the analysis. Data preprocessing involves a series of refinement processes, such as deleting words from a large set of documents that may contaminate the analysis results and unifying words with similar meanings [20]. The data analyzed in this study were obtained from a knowledge document of soccer psychology, and words (except for keywords that imply the meaning of the subject area of each paper) were analyzed. For example, from the words that were initially identified, we deleted words that describe the methodological unit of the paper, such as “purpose”, “research”, “method”, “result”, “discussion”, and “conclusion”, and adverbs and adjectives that are not keywords related to the actual research topic, such as “tomorrow”, “meanwhile”, “recently”, “always”, and “although”. In addition, words that have the same meaning but are recognized as separate keywords due to slight differences, including differences in expression, were combined, which may have affected the analysis results. Thus, the task of unifying multiple keywords with the same meaning was performed. All of this was carried out by the authors of this study, who are experts in natural language processing and topic modeling.

Meanwhile, among the representative keywords that describe the research topic area, a potential problem is that the representativeness of the area is excessively high and only the keyword influences the overall result. In this study, keywords such as “soccer” and “football” appeared as keywords in all papers at the time of the initial search. Thus, even if they do not appear in individual clusters of the topic model to be analyzed, they are sufficiently recognizable as basic topic areas. As an indicator to determine the presence of such words, TF-IDF(Term Frequency-Inverse Document Frequency) is a formula that calculates the number of words that appear universally in all documents and words that appear frequently in specific documents (i.e., keywords that are representative of the topic area) [21]. TF-IDF indexes above 0.3 are generally deleted [22]. After conducting these preprocessing steps, we refined the final 1430 words and utilized them for the analysis.

### 2.3. Topic Modeling

Various types of algorithms can be used for topic modeling, depending on the calculation method used, including individual analysis techniques. In this study, we used the latent Dirichlet allocation (LDA) technique. LDA is generally adopted when the topic areas across documents have a strong tendency to be reduced to a single contact point; it is also used to derive topic modeling results for knowledge technology documents that contain specific domain knowledge such as papers, news, and patents [23,24]. The parameter values of alpha, beta, and the number of topics must be arbitrarily specified in the topic modeling analysis settings to derive analysis results through topic modeling. However, both alpha and beta range from 0.01 to 0.99 and, in some cases, they can be divided into thousands of digits [25]. Furthermore, determining the number of cases, calculating the number of topics, and finding the optimal topic organization can be very difficult. These difficulties can be overcome by utilizing the coherence coefficient to find the optimal number of topics and keywords within topics [26].

**Figure 1:**
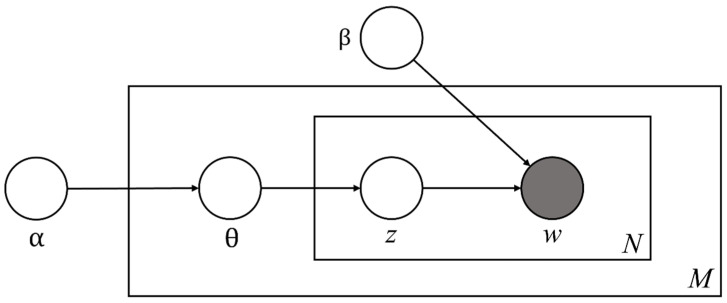
Probabilistic graphical model of latent Dirichlet allocation. α: a parameter that represents the Dirichlet prior for the document topic distribution; β: a parameter that represents the Dirichlet for the word distribution; θ: a vector for topic distribution over a document d; *z*: a topic for a chosen word in a document; *w*: specific words in *N*; *M*: the length of documents; *N*: the number of words in the document.

The coherence coefficient is a virtual proxy for the number of cases with the alpha, beta, and number of topics settings described above, and it adopts the number of cases with coherence values close to 1. In this study, the number of cases for alpha and beta values was set to 0.01~0.99 to consider the optimal number of topics and parameters for topic modeling results. The number of topics varied from seven to twenty, considering the total number of papers, and the consistency index was calculated for the number of all cases. As a result, the consistency index was very high (0.921) when the number of topics was fifty (alpha: 0.01, beta: 0.1). Therefore, by comparing the topic composition according to the number of topics, we derived core topics that show a high density of keyword composition in any setting and variable topics that indicate changes depending on the setting, and suggested the meaning and implications of the subject area implied by each topic. Figure 1 shows the conceptual model of LDA topic modeling utilized in this study.

### 2.4. Change in Topics by Period

This study analyzed the growth and decline of the topic area over time by analyzing the frequency and share of articles on psychological-related soccer research over a 33-year period (1990–2022). Topics were segregated based on discernible patterns of growth or decline over the specified time. A linear regression model was applied utilizing SPSS 23.0 (IBM Corp, Armonk, New York, NY, USA), with the timeline serving as the independent variable and the frequency and proportion of each topic acting as the dependent variables.

The results were subsequently interpreted using a systematic, two-step approach. First, after linear regression analysis, if the Durbin–Watson statistic was within the range of 1.5 to 2.5, it was considered a suitable regression model, and the regression coefficient and significance were checked [27,28]. Topics were categorized as hot if the coefficient was positive (+) and statistically significant, warm if the coefficient was positive (+) but lacked statistical significance, cold if the standardized regression coefficient was negative (−) with statistical significance, or cool if the coefficient was negative (−) but not statistically significant.

## 3. Results

### 3.1. Topic Modeling

The topic modeling results are shown in Table 1. The clustered interest of Topic 1 appears to be related to soccer motor skill training, specifically focusing on leg work; accuracy and speed with the ball; and motor tasks related to foot ability, balance, knee improvement, and coordination. Topic 2 was clustered and was related to soccer training and performance, particularly regarding physical characteristics and requirements. Topic 3 was clustered and demonstrated the importance of coaching and leadership by measuring and analyzing the strong relationships that effective coaching and leadership create, the establishment of clear roles and responsibilities, the creation of a supportive atmosphere, satisfaction, motivation, efficacy, cohesion, support, style, atmosphere, commitment, youth, and autonomy. 

**Figure 2:**
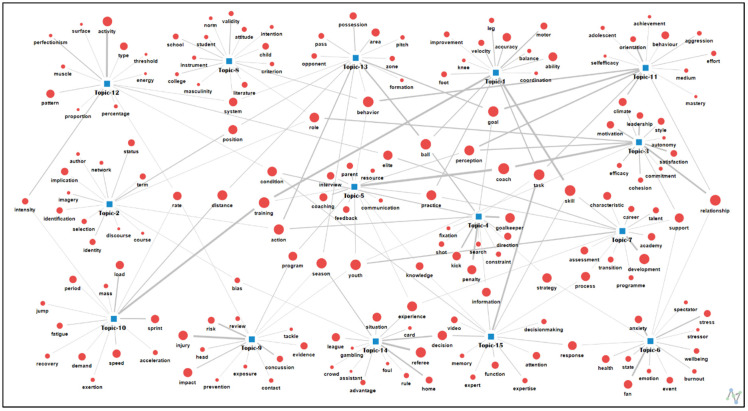
Knowledge structure by topic modeling.

Topic 4 focused on the role of goalkeepers in soccer, including their behavior and strategies when faced with penalty kicks and shots on goal. Topic 5 was clustered around the coaching process (encompassing feedback, practice, training, and communication, among other aspects) and considered the crucial role of coaches in developing youth programs and their support towards parents. Topic 6 was clustered with keywords related to psychological aspects of sports, such as anxiety, stress, burnout, and emotions, as well as strategies and support for coping with stressors and events. Topic 7 was clustered into keywords related to youth athlete development in elite sports academies, including talent and career assessment processes, transition, and support. Topic 8 was clustered with keywords related to the validity and reliability of measures and instruments used to assess soccer student-athlete behavior, including standards, norms, and attitudes. Topic 9 was clustered with keywords related to injuries in soccer—specifically regarding the risk of concussions and head impacts—and brain injury prevention and exposure review for adult, youth, and elite soccer players during the season. Topic 10 was clustered into keywords related to training load and physical demands, including fatigue, speed, acceleration, intensity, and recovery, which affect soccer players’ physical performance, including their sprinting, long-distance running, and jumping performance. Topic 11 was clustered with keywords related to soccer players’ goal orientations and behaviors, including perception and mastery, aggression, self-efficacy, and achievement in youth development. Topic 12 was clustered with keywords related to the pattern and intensity of physical activity, including perfectionism, energy, muscle, and threshold states in the soccer elite sport system. Topic 13 was considered to explore the different tactical formations used by soccer teams and how they affect gameplay. Topic 14 was clustered with keywords related to decision-making processes involving expert knowledge, information, and video analysis of technical functions, conditions, and reactions in soccer matches. A visualization of the keyword network for each topic is shown in Figure 2.

### 3.2. Trend Analysis by Topic Area

Each of the 14 clustered topics followed a detailed micro-classification of the soccer psychology domain. In the book “Football Psychology: From Theory to Practice”, Konter et al. (2019) organized the psychology of soccer into four macro-content areas, which were further organized into micro-disciplines [29]. Following this scheme, each topic was included in the subtopics of the four topic areas. The organization of topics in Konter’s (2019) book is as follows.

First, the “Motivation, Cognition, and Emotion” section focuses on soccer players’ motivation and psychological factors such as mood, grit, and mental strength. The topic modeling resulted in the inclusion of Topics 6 and 11, which are related to this topic area. 

Second, the “Coaching Essentials in Football” section covers the definition of a coach, competencies, leadership, effective coaching methods (e.g., communication), and psychological aspects of training and practice. The topic modeling resulted in the inclusion of Topics 1, 2, 3, 10, and 13, which are related to this topic area. Third, “Psychological Skills for Performance Development” covers various psychotechnical approaches to improving soccer performance, including the development of psychological skills for goalkeeper performance development. The topic modeling resulted in the inclusion of Topics 4, 12, and 14 which are related to this topic area. Fourth, the “Developing the young player in Football” section covers parental coaching, talent development, education and support on how to succeed, and prevention of negative psychological factors in developing young players into elite players. The topic modeling resulted in the inclusion of Topics 5, 7, 8, and 9, which are related to this topic area.

**Figure 3:**
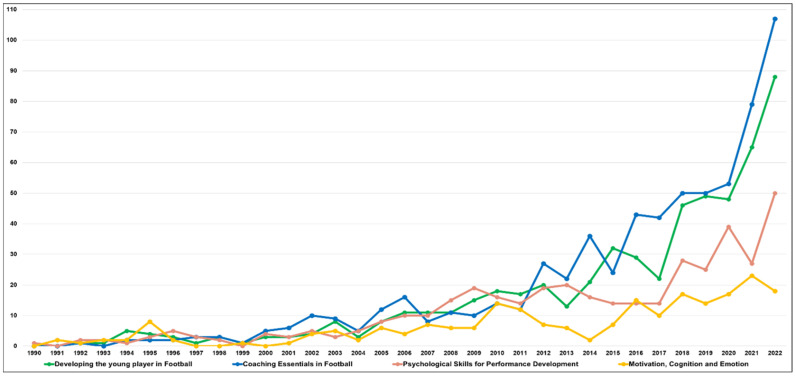
Frequency change for each subject area in football psychology.

We assessed the change in trends over time for each clustered topic area. The change in frequency of each topic area from 1990, when soccer psychology research was first identified, to 2022 is shown in Figure 3. All four topic areas experienced similar increases and decreases from the 1990s to the early 2000s. However, since the mid-2000s, the topic areas of “Coaching Essentials in Football” and “Developing the young player in Football” have experienced a steep increase in frequency. The frequencies of the topic areas of “Psychological Skills for Performance Development” and “Motivation, Cognition, and Emotion” have also increased but with a more modest linear trend. 

The trend changes based on the frequency of each topic area shown in Figure 3 were evaluated by linear regression (Table 2). The Durbin–Watson values were 0.263 (Developing the young player in Football), 0.272 (Coaching Essentials in Football), 0.784 (Psychological Skills for Performance Development), and 0.759 (Motivation, Cognition, and Emotion), respectively, and were not classified. The trend change was not statistically validated, even though both the frequency and the change in the chart showed an upward linear trend. Therefore, the change in the share of each year of the topic area was explored, and the trend change was statistically validated (Table 3). 

**Figure 4:**
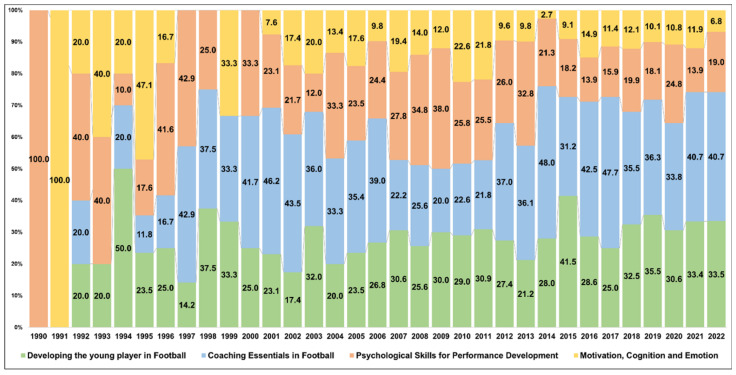
Changes in occupancy rate (%) of football psychology by subject area.

Figure 4 shows the change in the share of each topic area by year. The topic area “Developing the young player in Football” had a share of 20–30%, except in 1997 (14.2%) and 2015 (41.5%). The topic area of “Coaching Essentials in Football” was characterized by a minimal representation of 0–20% prior to 1996 and experienced a substantial surge after 1996. Notably, in 10 years, this topic area had a share of 40% or more, and its contribution peaked between 2010 and 2020. However, we observed a large variation in its share over time. 

Table 3 shows the results of the trend analysis of the occupancy rate of each topic area using linear regression. The topic area “Developing the young player in Football” had a Durbin–Watson value of 1.554, which is close to 2, meaning the trend could be validated through the regression coefficient. The regression coefficient was 0.470 (*p* = 0.006), which is a significant positive coefficient, indicating that this was a hot topic. The topic area “Essentials of Soccer Coaching” had a Durbin–Watson value of 0.908, which is not in the range of 1.5 to 2.5. Therefore, the trend was not statistically significant. The topic area “Psychological Skills for Performance Development” had a Durbin–Watson value of 1.981, which is close to 2, meaning the trend could be validated through the regression coefficient. The regression coefficient was −0.384, which is not statistically significant; thus, this topic area was categorized as cool. Finally, the topic area “Motivation, Cognition, and Emotion” had a Durbin–Watson value of 2.443, which is close to 2, meaning that the trend could be validated through the regression coefficient. The regression coefficient was −0.384 (*p* > 0.027), which indicates a significant positive coefficient; therefore, this topic was classified as cold.

## 4. Discussion

### 4.1. Topic Area 1: Developing the Young Player in Football

“Developing the young player in Football” is an area of research focusing on the importance of understanding the player development process in identifying and nurturing soccer talent. This topic area was organized into Topics 5, 7, 8, and 9. Topic 5 has explored how feedback from parents and coaches can influence youth development [30,31]. In addition, the most effective ways to provide feedback to young players have been investigated [32,33]. Approaches related to Topic 7 include how youth athletes could develop the skills and traits needed to succeed at the elite level, an exploration of effective strategies for identifying and nurturing talent in young athletes [34], and how coaches and programs can balance individual skill development with team success and cohesion [35,36]. Topic 8 explores the constructs of masculinity, attitudes, intentions, and behaviors within soccer-educational settings, the college or school level [37,38]. Furthermore, this topic aims to unravel how these norms might be manipulated by the prevalent school system and the post-pandemic sports culture [39,40]. This topic was investigated through a critical lens to understand the socio-cultural aspects of soccer and its effects on individual players’ behaviors. Topic 9 deeply investigates the pressing issue of injury risk, specifically focusing on concussions within the context of youth soccer. Research approaches related to this topic include the common types of brain-related injuries in soccer, including concussions, and how they can be prevented or managed [41,42]; methods that coaches and organizations can employ to mitigate the risk of concussion and head injury in youth soccer [43,44]; and evidence-based strategies that can be used to reduce the incidence of brain and head-related injuries in soccer players [45,46]. 

The process of effectively identifying and developing players with the potential for future success is becoming increasingly important [47]. The importance of these studies focusing on youth and adolescents is also evident in review studies related to athletic development. Kirkendall and Krustrup (2022) reported that among training programs research targeting women and all genders, a higher percentage of program interventions focused on youth and adolescent soccer players aged 6–18 years than adult soccer players [48]. However, effectively providing the necessary conditions for youth and young athletes to reach their potential takes more than just accumulating quantitative research. Studies that have attempted to address these issues can be found in this topic area. Psychological approaches and different training and support structures have been proposed to facilitate feedback and communication between coaches and parents at all levels, from the elite youth level onwards [49,50]. These research efforts have contributed to the consideration of more advanced communication methods and training systems for youth soccer players. In particular, the health and safety of athletes should be a top priority for youth sports teams and related organizations [51]. Part of the research in this topic area recognizes this importance and has contributed to the focus on “injuries” and related warnings and responses [52]. Furthermore, this research area has highlighted the importance of understanding the complex socio-cultural norms, especially masculinity, that permeate football at the educational level [53]. In some respects, these discourses have contributed to providing an opportunity to revisit policies and frameworks to ensure an inclusive and progressive environment for youth players.

The trends in this thematic area are a sharp increase in the frequency of research and a steady share after a certain period. After a certain period, the first paper in this topic area in the research period 1990–2022 was found in 1992. Although the frequency of research in this topic area fluctuates from year to year, there has been a steep increase since 2017, and the frequency has been increasing until now. The trend for increased frequency was not statistically significant; however, it was the only one of all topic areas to show a significant increase in share. An explosion in share was not observed by us either, but it was maintained steady in the 1990s, when the overall frequency of soccer psychology research was significantly lower. This suggests that this topic area can be expected to remain active according to the share regression model. Since the 1990s, the importance of psychological approaches in the development of youth players has been raised in various sports, including soccer [54]. Nevertheless, due to the lack of research approaches and infrastructure at the time, it seems to have been more discourse-driven than quantitative growth [55]. However, it is worth noting that, since the 2000s, there has been a growing consensus that the soccer development system for youth needs a major overhaul [56]. The impetus for this has come from the implications of most members of the system, including teams, coaches, players, and parents [57,58]. The market growth of the soccer sector and the business aspect of massive capital inflows have also given us the impetus to focus on the development system of youth elite sports [56,59]. This has allowed us to build an effective youth development system, which has been difficult to realize, and to support various research efforts to develop and improve the system [60]. This trend and flow in soccer area is interpreted by us as the reason for the growth in frequency and share of this study area. However, the psychological approach to youth development is far from complete. The sensitivity and the fearful nature of the youth generation to external stimuli are bound to be greatly influenced by changing times, societies, and systems. Therefore, we can cautiously predict that the field of psychological research with youth soccer players will continue to identify new research topics that can respond to new and anomalous paradigms.

### 4.2. Topic Area 2: Coaching Essentials in Football

“Coaching Essentials in Football” refers to research on coaching, which is an essential component of elite sports, including soccer. Research in this area explores the relationship between coaches and players and the effectiveness of different coaching styles and support structures. This topic area was organized into Topics 1, 2, 3, 10, and 13. Topic 1 addresses the importance of skill training for the motor tasks that soccer players need to perform. Relevant research has identified the most effective training methods and strategies for improving accuracy, speed, footwork, balance, and coordination [61,62,63]. Topic 2 keyword clusters show that studies have explored how the ‘coach’s status or role affects their identity and perception of their place in society [64,65], and how language and discourse can influence the formation and reinforcement of identity and bias [65,66]. Topic 3 research has examined how coaches can build effective relationships with athletes and how this can affect their motivation and performance [67]. Furthermore, studies on this topic have explored the common leadership styles in coaching and how they can affect team cohesion and success [68,69]. Topic 10 includes research examining how soccer coaches and players can effectively manage training load to optimize performance and reduce the risk of injury or burnout, the physiological and psychological factors that contribute to fatigue during training or competition, and how this fatigue can be mitigated [70,71], and how players and coaches can balance training and competition demands with the need for rest and recovery [72,73]. Topic 13 includes studies exploring how ball possession and distance affect the likelihood of scoring [74,75]; how different formations and positioning affect player behavior, team chemistry, and performance during a match; and the key factors that determine successful passes in soccer and how they vary across different areas and zones of the field [76,77]. 

Consistent with the sport-wide notion that coaches’ efforts to improve their coaching skills are fundamental to all sports and should be taken seriously, soccer psychology is one of the most active areas of coaching research [78]. The Essentials of Soccer Coaching emphasizes the multifaceted nature of soccer coaching. From technical development and psychological understanding to tactical acumen and load management, effective coaching brings together a range of areas to demonstrate the role of coaching in individual player and team achievement [79]. It has been reaffirmed that coaching is based on precise training to improve key skills such as accuracy, speed, footwork, balance, and coordination in player play. In addition, it has contributed to the provision of a range of applied coaching strategies in the tactical aspects of ball possession, positioning, passing, maneuvering, and formation to create scoring opportunities and defending effectively [80]. Insights into the application of social dynamics for efficient physical recovery and team cohesion, not just to improve individual and team performance, have been observed through various studies within this thematic area [81].

The research area “Coaching Essentials in Soccer” ranks high on the list of research areas in bibliographic analyses of soccer psychology research [48,82]. During the 1990–2022 research period, the first paper on this topic area was published in 1992. This topic area has increased in frequency, albeit with year-to-year variations, with a steep upward trend since 2017. No statistical significance was found in the trend classification for the increase in frequency, and no significant statistical trend classification was performed in the regression model for share; therefore, further exploration and measurement is needed to identify trends in this topic area. However, this topic has the highest frequency, a generally high share, and is an important topic for many studies. In particular, the recent surge in soccer training research is likely due to the relative increase in studies of training environments due to the COVID-19 pandemic, which has limited evaluation of on-field performance [83,84]. While statistical significance is difficult to ascertain due to the erratic nature of the soccer coaching research field due to these internal and external factors, the highest publication and rapid increase in frequency is the most widespread research area. The post-COVID-19 changes can also be noted, as coaching and training-related research topics have been influenced by the overall research environment in sport during the COVID-19 pandemic compared to pre-COVID-19 [85]. In the post-pandemic era, it is worth noting whether the coaching and training environment has returned to pre-pandemic norms or whether new paradigms are being applied [86]. This will be an opportune time to observe and analyze the flow of research on soccer coaching and training in the post-pandemic environment, as well as to establish creative research directions in response to that flow.

### 4.3. Topic Area 3: Psychological Skills for Performance Development

The topic area “Psychological Skills for Performance Development” addressed psychological approaches to soccer performance development, including the intervention of coaching but focusing on psychological skills. This topic area was organized into Topics 4, 12, and 14. Topic 4 has translated into topics exploring the strategies goalkeepers use to effectively defend against penalty kicks [87,88], how goalkeepers can improve their ability to predict the direction and force of shots [89,90], and the physical and mental conditions required for optimal goalkeeping performance, and the strategy for developing and maintaining this [91,92]. Topic 12 is a cluster including psychological skills approaches that have been attempted to effectively harness perfectionism, which has advantages and disadvantages. Several research areas have addressed these issues, including the optimization of perfectionist tendencies through psychological skills training and the development and validation of psychological and physical training programs that consider energy metabolism, thresholds, and muscle loading for effective intensity setting [93,94]. Topic 14 explores the key factors that influence player decision making when carrying out the complex tasks presented in a soccer game; how soccer players’ expertise and competence develop over time; the roles of factors such as memory, attention, and knowledge in this process [95,96], and the most effective strategies for improving decision making in tight game situations [97,98]. Furthermore, research has explored ways to improve soccer players’ decision making and skill development using new technologies such as virtual reality and cognitive training programs [99,100]. 

One of the proven facts in this area of research is the ambivalence about perfectionism in soccer players. This is evidenced across almost all sports, and soccer is no exception [101,102]. This area of research has contributed to the demonstration of psycho-skills training for optimal perfectionism that can lead to excellence under pressure. In addition to physical skills, a player’s memory, attention, and domain knowledge are important factors in making split-second decisions during training or matches [103]. This highlights the importance of psycho-skills training for players, including goalkeepers, to clearly identify their roles and make effective decisions for each position [104]. In conclusion, the “Psychological Skills for Performance Development” topic area underscores the intricate interplay between the mind and body in soccer. Research within this topic area has contributed to the development and application of context-specific psychological skills training, from reaching an optimal state of perfectionism to effectively accomplishing the unique challenges faced by players in different positions. In addition, this area of research has shown that the correct decision making of players and all other units that make up a soccer match improves the quality of the game. On the other hand, advanced technologies such as virtual reality can effectively enhance the psychological and logical state of soccer players, which adds to the diversity of this research area.

Frequency measures and significance tests in the regression model show that the number of studies on this topic published each year is not decreasing. However, as the overall research area of soccer psychology has increased, the share of this topic area has decreased somewhat over time. For this reason, it appears to be a somewhat stagnant research area according to the share regression model. However, this is more related to the relative activation of other research areas rather than a decrease in interest in this topic area. Researchers such as Mills et al. (2012) suggested that athletes do not need to possess all of the skills recommended in the talent development literature to develop at an elite level [79]. Instead, the authors explain that some athletes can make the transition successfully even in the absence of some psychological skills [79]. In other words, it cannot be assumed that applying psychological skills to a talented athlete will automatically make them an elite athlete, as there are a variety of contextual and environmental factors, such as finances, health, and other personal circumstances, that can have a significant impact on an athlete’s likelihood of reaching an elite level [105]. It is because these factors have been ignored that various review studies of psychological skills training have been criticized for generalizing their findings by focusing on athletes at a specific developmental stage [106]. These limitations are also thought to have contributed to the lack of exponential growth in the number of studies and applications of psychological skills training. However, some studies have emphasized the importance of psychological skills training in the juvenile system. While psycho-skills training at the adult level is dependent on individual differences and influenced by contextual variables, it is important to note that psycho-skills training systems for youth should not be neglected [107]. According to several authors, perspectives on the adherence and success of these types of programs would be fundamentally different if they were targeted at younger athletes instead of focusing on more experienced athletes [108]. Furthermore, research on the development and validation of psycho-skills training across different ages and stages of athlete development would be valuable future work.

### 4.4. Topic Area 4: Motivation, Cognition, and Emotion

The topic area “Motivation, Cognition, and Emotion” was organized into topics that examined and evaluated psychological traits that occur in different situations experienced by soccer players. This topic area was organized into Topics 6 and 11. Topic 6 aims to understand professional soccer players’ experiences with burnout, with a focus on potential causes, such as overtraining, personal stressors, or the pressures of professional sports. Moreover, matches were determined and investigations were conducted to observe how stress and anxiety levels might affect players’ performance [109,110]. Research on this topic has also shown how relationships with coaches, teammates, and others influence players’ stress levels and mental health [111,112]. Topic 11 was examined to determine how players’ goal orientation (e.g., mastery goals versus performance goals) influences their performance on the field. This topic explores perceptions of self-efficacy among soccer players and its impact on performance [113,114]. This topic also explores how the team atmosphere affects players’ aggression and effort [114,115]. 

Given the different goal orientations of athletes, the research identified in this topic area provides insight into considering whether soccer players are more focused on mastery goals or performance goals [116,117], which will contribute to the development of personalized motivational strategies and help coaches and trainers provide more effective coaching [118]. In addition, this knowledge will lead to the development of interventions and strategies to support soccer players’ mental health and prevent issues such as burnout, depression, and severe anxiety [119]. It is also important to note that good mental health in individual players can set the stage for a positive team culture [117]. Overall, this topic has contributed to a more holistic understanding of soccer players’ mental health, which can improve the way players are coached, the way clubs are managed, and the way mental health is considered in soccer.

The first paper in the database collected for this study, from 1990, was found within this topic area. The frequency of studies increases and decreases every year, and even the share has been gradually decreasing since 2010. The regression model for the decrease in share was found to be statistically significant. Overall, we can cautiously diagnose that research in this topic area of soccer psychology has stagnated somewhat. This topic area is largely composed of studies that have used questionnaires to collect and evaluate data. However, most research in elite sports, including soccer, is increasingly focused on direct application to the field [120]. For a long time now, much of the research in soccer psychology has focused on practical approaches to developing physical, tactical, and technical factors with a psychological component [121]. These topics receive a lot of attention because they can have a big impact on immediate performance improvement, which lends itself to real-world soccer applications [122]. Furthermore, these field-oriented studies inevitably have a high proportion of interdisciplinary approaches, such as psycho-physiological, psycho-performance, and psycho-physical approaches [123,124]. Therefore, there are relatively few studies exploring psychological factors alone. Nevertheless, the exploration of psychological factors in players provides a foundation for the development of new psycho-skills training, psychophysiological programs in training and their application on the field [125]. Therefore, it is important to develop instruments with high reliability and validity to measure psychological factors in this area, and this effort is ongoing [126,127,128]. From the foregoing, it can be cautiously predicted that research in this topic area will continue to be of value through its multidisciplinary approach to topics across soccer psychology, including training, psychological skill development, and performance development in youth and adult populations.

## 5. Conclusions

This study aimed to identify the knowledge structure of and research trends in soccer research from 1990–2022. Topic modeling resulted in 14 topics. The topic area “Essential Coaching in Soccer” was the most frequent, but not statistically trending. However, coaching, including training, is expected to continue to be an important research topic as it is a key requirement for success in the highly competitive world of elite soccer. Interest in the research area of “psychological skills for performance development” has waned in recent years, possibly due to the dominance of other subjects rather than a lack of interest. There are a number of cutting-edge interventions and problem-solving attempts in this area, providing opportunities for qualitative and quantitative expansion. “Motivation, cognition, and emotion” is a vastly underappreciated topic area in soccer psychology. This may be due to the fact that recent soccer research has emphasized the importance of rapid on-field application, resulting in fewer attempts at survey-based psychological assessment. However, measuring psychological factors contributes to the study of soccer psychology through new methodologies and theoretical backgrounds. Recognizing the important role that psychological factors play in players’ performance and mental management and providing new research directions and approaches with direct field application will advance soccer psychology research. Based on the theoretical basis of previous studies, this study searched for articles in WOS alone, but it is necessary to conduct an approach to identify the knowledge structure of soccer-psychology research topics that exist in a wider range of indexes and databases. Therefore, insights from an integrative perspective of data collection and analysis of academic databases including WOS, Scopus, and SportDISCUS are proposed as a future research direction.

## Figures and Tables

**Table 1:** Results of topic modeling in soccer psychology.

Topic 1	Topic-2	Topic 3	Topic 4	Topic 5
skill	position	coach	goalkeeper	coach
training	identity	relationship	penalty	coaching
motor	status	leadership	kick	parent
task	network	satisfaction	ball	feedback
leg	imagery	motivation	condition	perception
practice	term	perception	action	practice
ball	selection	role	goal	youth
accuracy	identification	efficacy	direction	program
velocity	role	cohesion	shot	interview
foot	bias	support	task	training
ability	action	style	search	experience
balance	author	relation	information	behavior
knee	course	commitment	strategy	communication
improvement	implication	coaching	constraint	knowledge
coordination	discourse	autonomy	fixation	resource
**Topic 6**	**Topic 7**	**Topic 8**	**Topic 9**	**Topic 10**
anxiety	development	validity	injury	training
stress	youth	student	risk	load
health	elite	behavior	concussion	sprint
state	skill	instrument	impact	distance
burnout	academy	college	head	fatigue
wellbeing	talent	school	program	speed
emotion	career	child	youth	rate
support	practice	criterion	prevention	acceleration
response	transition	role	exposure	jump
relationship	assessment	norm	review	period
stressor	process	masculinity	season	mass
mood	program	attitude	tackle	demand
strategy	experience	intention	rate	exertion
depression	support	system	evidence	intensity
behavior	characteristic	literature	contact	recovery
**Topic 11**	**Topic 12**	**Topic 13**	**Topic 14**	
goal	activity	ball	task	
behavior	intensity	goal	decision	
orientation	perfectionism	action	expertise	
perception	elite	possession	information	
climate	pattern	season	skill	
task	surface	position	function	
relationship	type	distance	condition	
aggression	energy	pass	response	
effort	muscle	area	process	
mastery	percentage	zone	decision making	
self-efficacy	threshold	pitch	expert	
behavior	condition	behavior	memory	
medium	proportion	system	attention	
adolescent	rate	opponent	video	
achievement	system	formation	knowledge	

**Table 2:** Trend classification using regression analysis of frequency change by topic area.

Topic Area	Topic No.	Durbin–Watson	Coefficient	t-Value	*p*-Value	Hot/Cold
Developing the young player in Football	Topic 5	0.263	0.834	8.431	0.001	-
	Topic 7					
	Topic 8					
	Topic 9					
Coaching Essentials in Football	Topic 1	0.272	0.836	8.498	0.001	-
	Topic 2					
	Topic 3					
	Topic 10					
	Topic 13					
Psychological Skills for Performance Development	Topic 4	0.784	0.865	9.616	0.001	-
	Topic 12					
	Topic 14					
Motivation, Cognition, and Emotion	Topic 6	0.759	0.816	7.850	0.001	-
	Topic 11					

**Table 3:** Trend classification using regression analysis of occupancy rate change by topic area.

Topic Area	Topic No.	Durbin–Watson	Coefficient	t-Value	*p*-Value	Hot/Cold
Developing the young player in Football	Topic 5	1.554	0.470	2.961	0.006	Hot
	Topic 7					
	Topic 8					
	Topic 9					
Coaching Essentials in Football	Topic 1	0.908	0.585	4.019	0.001	
	Topic 2					
	Topic 3					
	Topic 10					
	Topic 13					
Psychological Skills for Performance Development	Topic 4	1.981	−0.325	−1.912	0.065	Cool
	Topic 12					
	Topic 14					
Motivation, Cognition, and Emotion	Topic 6	2.443	−0.384	−2.31	0.027	Cold
	Topic 11					

## Data Availability

The data presented in this study are available on request from the corresponding authors.

## References

[1] Kotrba V. (2019). Direct preferences of sports fans: Is there a superstar effect in the fantasy league?. J. Behav. Exp. Econ..

[2] Hammami A., Gabbett T.J., Slimani M., Bouhlel E. (2018). Does small-sided games training improve physical fitness and team-sport-specific skills? A systematic review and meta-analysis. J. Sports Med. Phys. Fit..

[3] Bouguezzi R., Chaabene H., Negra Y., Moran J., Sammoud S., Ramirez-Campillo R., Granacher U., Hachana Y. (2019). Effects of jump exercises with and without stretch-shortening cycle actions on components of physical fitness in prepubertal male soccer players. Sport Sci. Health.

[4] Villalonga T., Garcia-Mas A., de las Heras R., Buceta C., Smith R.E. (2015). Creation and Tasks of a Sport Psychology Department in a Professional Soccer Club. Rev. Psicol. Deporte..

[5] Dean F., Kavanagh E., Wilding A., Rees T. (2022). An Examination of the Experiences of Practitioners Delivering Sport Psychology Services within English Premier League Soccer Academies. Sports.

[6] Hirose K., Meijen C. (2022). An exploration of elite Japanese female footballers’ acute cultural transition experiences in Europe. Sci. Med. Footb..

[7] Lavallee D. (2005). The Effect of a Life Development Intervention on Sports Career Transition Adjustment. Sport Psychol..

[8] Holt J.E., Kinchin G., Clarke G. (2012). Effects of peer-assessed feedback, goal setting and a group contingency on performance and learning by 10–12-year-old academy soccer players. Phys. Educ. Sport Pedagog..

[9] Duncan M.J., Weldon A., Barnett L.M., Lander N. (2022). Perceptions and practices of fundamental movement skills in grassroots soccer coaches. Int. J. Sports Sci. Coach..

[10] Klarin A. (2019). Mapping product and service innovation: A bibliometric analysis and a typology. Technol. Forecast. Soc. Chang..

[11] Scaccia J.P. (2021). Examining the concept of equity in community psychology with natural language processing. J. Community Psychol..

[12] Hirschberg J., Manning C.D. (2015). Advances in natural language processing. Science.

[13] Robaldo L., Villata S., Wyner A., Grabmair M. (2019). Introduction for artificial intelligence and law: Special issue “natural language processing for legal texts”. Artif. Intell. Law.

[14] Vayansky I., Kumar S.A.P. (2020). A review of topic modeling methods. Inf. Syst..

[15] Ma Y., Chen D., Wang T., Li G., Yan M. (2022). Semi-supervised partial label learning algorithm via reliable label propagation. Appl. Intell..

[16] Bruner M.W., Erickson K., Wilson B., Côté J. (2010). An appraisal of athlete development models through citation network analysis. Psychol. Sport Exerc..

[17] Cheng X., Shuai C., Liu J., Wang J., Liu Y., Li W., Shuai J. (2018). Topic modelling of ecology, environment and poverty nexus: An integrated framework. Agric. Ecosyst. Environ..

[18] Suominen A., Toivanen H. (2015). Map of science with topic modeling: Comparison of unsupervised learning and human-assigned subject classification. J. Assoc. Inf. Sci. Technol..

[19] Corcoran C.M., Mittal V.A., Bearden C.E., Gur R.E., Hitczenko K., Bilgrami Z., Savic A., Cecchi G.A., Wolff P. (2020). Language as a biomarker for psychosis: A natural language processing approach. Schizophr. Res..

[20] Zhao Y.-F., Fu Z.-G., Chen F. Research on Big data Preprocessing Technology of Thermal System. Proceedings of the 2nd Annual International Conference on Energy, Environmental & Sustainable Ecosystem Development (EESED 2016).

[21] Tighe P.J., Sannapaneni B., Fillingim R.B., Doyle C., Kent M., Shickel B., Rashidi P. (2020). Forty-two Million Ways to Describe Pain: Topic Modeling of 200,000 PubMed Pain-Related Abstracts Using Natural Language Processing and Deep Learning–Based Text Generation. Pain Med..

[22] Kamyab M., Liu G., Adjeisah M. (2021). Attention-Based CNN and Bi-LSTM Model Based on TF-IDF and GloVe Word Embedding for Sentiment Analysis. Appl. Sci..

[23] Jelodar H., Wang Y., Yuan C., Feng X. (2019). Latent Dirichlet Allocation (LDA) and Topic modeling: Models, applications, a survey. Multimed. Tools Appl..

[24] Tian C., Zhang J., Liu D., Wang Q., Lin S. (2022). Technological topic analysis of standard-essential patents based on the improved Latent Dirichlet Allocation (LDA) model. Technol. Anal. Strat. Manag..

[25] Vorontsov K., Potapenko A., Plavin A. Additive Regularization of Topic Models for Topic Selection and Sparse Factorization. Proceedings of the Statistical Learning and Data Sciences: Third International Symposium, SLDS 2015.

[26] O’callaghan D., Greene D., Carthy J., Cunningham P. (2015). An analysis of the coherence of descriptors in topic modeling. Expert Syst. Appl..

[27] Durbin J., Watson G.S. (1971). Testing for Serial Correlation in Least Squares Regression. III. Biometrika.

[28] Seo Y., Kim K., Kim J.-S. (2021). Trends of Nursing Research on Accidental Falls: A Topic Modeling Analysis. Int. J. Environ. Res. Public Health.

[29] Konter E., Beckmann J., Loughead T.M. (2019). Football Psychology: From Theory to Practice.

[30] Kwon J., Elliott S., Velardo S. (2020). Exploring perceptions about the feasibility of educational video resources as a strategy to support parental involvement in youth soccer. Psychol. Sport Exerc..

[31] Kim S., Connaughton D.P. (2021). Soccer, concussions, and safety: Perceptions of parents of youth soccer participants. J. Saf. Res..

[32] Høigaard R., Haugen T., Johansen B.T., Giske R. (2017). Team identity in youth soccer: The role of coaches’ feedback patterns and use of humour. Int. J. Sports Sci. Coach..

[33] Yiannaki C., Carling C., Collins D. (2018). Futsal as a potential talent development modality for soccer—A quantitative assessment of high-level soccer coach and player perceptions. Sci. Med. Footb..

[34] Yiannaki C., Carling C., Collins D. (2017). Could futsal hold the key to developing the next generation of youth soccer players?. Sci. Med. Footb..

[35] Hendry D.T., Williams A.M., Hodges N.J. (2018). Coach ratings of skills and their relations to practice, play and successful transitions from youth-elite to adult-professional status in soccer. J. Sports Sci..

[36] Agustí D., Ballester R., Juan-Blay J., Taylor W.G., Huertas F. (2020). The Academic Background of Youth Soccer Coaches Modulates Their Behavior During Training. Front. Psychol..

[37] Adams A. (2011). “Josh Wears Pink Cleats”: Inclusive Masculinity on the Soccer Field. J. Homosex..

[38] Kennedy K.W., Steinfeldt J.A., Eppich A., Halterman A.W., Kinne A., Zakrajsek R.A. (2021). Football players’ beliefs about masculinity, affection between men, and gay teammates. Psychol. Men Masculinities.

[39] Van Raalte J.L., Brewer B.W., Brewer D.D., Linder D.E. (1992). NCAA Division II College Football Players’ Perceptions of an Athlete Who Consults a Sport Psychologist. J. Sport Exerc. Psychol..

[40] Jin G. (2022). The Influence of Football Exercise on the Rehabilitation of Chinese College Students with Mental Illness. Psychiatr. Danub..

[41] Abbas K., Shenk T.E., Poole V.N., Robinson M.E., Leverenz L.J., Nauman E.A., Talavage T.M. (2015). Effects of Repetitive Sub-Concussive Brain Injury on the Functional Connectivity of Default Mode Network in High School Football Athletes. Dev. Neuropsychol..

[42] Colantonio A. (2020). Beyond football: Intimate partner violence and concussion/brain injury. Can. Psychol. Can..

[43] A Morgan E., Johnson S.T., E Bovbjerg V., Norcross M.F. (2017). Associations between player age and club soccer coaches’ perceptions of injury risk and lower extremity injury prevention program use. Int. J. Sports Sci. Coach..

[44] Terry D.P., Mewborn C.M., Miller L.S. (2019). Repeated Sport-Related Concussion Shows Only Minimal White Matter Differences Many Years After Playing High School Football. J. Int. Neuropsychol. Soc..

[45] Rusciano A., Corradini G., Stoianov I. (2017). Neuroplus biofeedback improves attention, resilience, and injury prevention in elite soccer players. Psychophysiology.

[46] Yao Z.-F., Sligte I.G., Moreau D., Hsieh S., Yang C.-T., Ridderinkhof K.R., Muggleton N.G., Wang C.-H. (2020). The brains of elite soccer players are subject to experience-dependent alterations in white matter connectivity. Cortex.

[47] Murr D., Raabe J., Höner O. (2017). The prognostic value of physiological and physical characteristics in youth soccer: A systematic review. Eur. J. Sport Sci..

[48] Kirkendall D.T., Krustrup P. (2021). Studying professional and recreational female footballers: A bibliometric exercise. Scand. J. Med. Sci. Sports.

[49] Goldstein J.D., Iso-Ahola S.E. (2008). Determinants of Parents’ Sideline-Rage Emotions and Behaviors at Youth Soccer Games. J. Appl. Soc. Psychol..

[50] Fleming D.J.M., Dorsch T.E., Dayley J.C. (2022). The mediating effect of parental warmth on the association of parent pressure and athlete perfectionism in adolescent soccer. Int. J. Sport Exerc. Psychol..

[51] Neumann N.D., Brauers J.J., Van Yperen N.W., Hasselman F., Den Hartigh R.J.R. (2023). Detecting early warning signals of injuries and health problems in elite youth soccer players. J. Sport Exercise Psy..

[52] Hennrikus W.L., Shaw B.A., Gerardi J.A., Ward A. (1998). A re-evaluation of injuries in youth soccer. Pediatrics.

[53] Adams A., Kavanagh E. (2017). Inclusive ideologies and passive performances: Exploring masculinities and attitudes toward gay peers among boys in an elite youth football academy. J. Gend. Stud..

[54] Stratton G. (2004). Youth Soccer: From Science to Performance.

[55] Inklaar H. (1994). Soccer Injuries. Sports Med..

[56] Gammelsæter H., Jakobsen S.-E. (2008). Models of Organization in Norwegian Professional Soccer. Eur. Sport Manag. Q..

[57] Moore M., Blom L., Califano K., Hussey K., Farello A., Vasiloff O., Gabler T., Sullivan M. (2021). Redesigning a Youth Soccer Program: Holistic Development of Athletes. Child Adolesc. Soc. Work. J..

[58] Eckardt V.C., Dorsch T.E., Lobinger B.H. (2021). Parents’ competitive stressors in professional German youth soccer academies: A mixed-method study. Psychol. Sport Exerc..

[59] Cocco A.R., Spencer T.C. (2019). Path to the Pros: How Major League Soccer Is Revolutionizing Youth Player Development in the United States.

[60] Mullin B.J., Hardy S., Sutton W. (2014). Sport Marketing.

[61] Zhongfan L., Inomata K., Takeda T. (2002). Soccer Players’ and Closed-Skill Athletes’ Execution of a Complex Motor Skill. Percept. Mot. Ski..

[62] Egan C.D., Verheul M.H.G., Savelsbergh G.J.P. (2007). Effects of Experience on the Coordination of Internally and Externally Timed Soccer Kicks. J. Mot. Behav..

[63] Ren Y., Wang C., Lu A. (2022). Effects of perceptual-cognitive tasks on inter-joint coordination of soccer players and ordinary college students. Front. Psychol..

[64] Zucchermaglio C. (2005). Who wins and who loses: The rhetorical manipulation of social identities in a soccer team. Group Dyn. Theory, Res. Pr..

[65] Adams A., Anderson E., McCormack M. (2010). Establishing and Challenging Masculinity: The Influence of Gendered Discourses in Organized Sport. J. Lang. Soc. Psychol..

[66] Cocking C., Drury J. (2013). Talking about Hillsborough: ‘Panic’ as Discourse in Survivors’ Accounts of the 1989 Football Stadium Disaster. J. Community Appl. Soc. Psychol..

[67] Myers N.D., Feltz D.L., Maier K.S., Wolfe E.W., Reckase M.D. (2006). Athletes’ Evaluations of Their Head Coach’s Coaching Competency. Res. Q. Exerc. Sport.

[68] Høigaard R., Jones G.W., Peters D.M. (2008). Preferred Coach Leadership Behaviour in Elite Soccer in Relation to Success and Failure. Int. J. Sports Sci. Coach..

[69] Keatlholetswe L., Malete L. (2019). Coaching Efficacy, Player Perceptions of Coaches’ Leadership Styles, and Team Performance in Premier League Soccer. Res. Q. Exerc. Sport.

[70] Haddad M., Chaouachi A., Wong D.P., Castagna C., Hambli M., Hue O., Chamari K. (2013). Influence of fatigue, stress, muscle soreness and sleep on perceived exertion during submaximal effort. Physiol. Behav..

[71] Coutinho D., Gonçalves B., Wong D.P., Travassos B., Coutts A.J., Sampaio J. (2018). Exploring the effects of mental and muscular fatigue in soccer players’ performance. Hum. Mov. Sci..

[72] Redkva P.E., da Silva S.G., Paes M.R., Dos-Santos J.W. (2016). The Relationship Between Coach and Player Training Load Perceptions in Professional Soccer. Percept. Mot. Ski..

[73] E Swallow W., Skidmore N., Page R.M., Malone J.J. (2020). An examination of in-season external training load in semi-professional soccer players: Considerations of one and two match weekly microcycles. Int. J. Sports Sci. Coach..

[74] Shafizadeh M., Lago-Penas C., Gridley A., Platt G.K. (2014). Temporal Analysis of Losing Possession of the Ball Leading to Conceding a Goal: A Study of the Incidence of Perturbation in Soccer. Int. J. Sports Sci. Coach..

[75] Nunes N.A., Gonçalves B., Coutinho D., Nakamura F.Y., Travassos B. (2020). How playing area dimension and number of players constrain football performance during unbalanced ball possession games. Int. J. Sports Sci. Coach..

[76] Clemente F.M., Couceiro M.S., Martins F.M.L., Mendes R., Figueiredo A.J. (2013). Measuring Tactical Behaviour Using Technological Metrics: Case Study of a Football Game. Int. J. Sports Sci. Coach..

[77] Fernandes T., Camerino O., Castañer M. (2021). T-Pattern Detection and Analysis of Football Players’ Tactical and Technical Defensive Behaviour Interactions: Insights for Training and Coaching Team Coordination. Front. Psychol..

[78] Gómez M.A., Lago-Peñas C., Gómez M.-T., Jimenez S., Leicht A.S. (2021). Impact of elite soccer coaching change on team performance according to coach- and club-related variables. Biol. Sport.

[79] Mills A., Butt J., Maynard I., Harwood C. (2012). Identifying factors perceived to influence the development of elite youth football academy players. J. Sports Sci..

[80] Branquinho L., Ferraz R., Marques M.C. (2021). 5-a-Side Game as a Tool for the Coach in Soccer Training. Strength Cond. J..

[81] Erikstad M.K., Johansen B.T., Johnsen M., Haugen T., Côté J. (2021). “As Many as Possible for as Long as Possible”—A Case Study of a Soccer Team That Fosters Multiple Outcomes. Sport Psychol..

[82] Cefis M. (2022). Football analytics: A bibliometric study about the last decade contributions. Electron. J. Appl. Stat..

[83] Partington M., Cushion C. (2011). An investigation of the practice activities and coaching behaviors of professional top-level youth soccer coaches. Scand. J. Med. Sci. Sports.

[84] Jones B., Eather N., Miller A., Morgan P.J. (2023). Evaluating the impact of a coach development intervention for improving coaching practices and player outcomes in football: The MASTER Coaching randomised control trial. Phys. Educ. Sport Pedagog..

[85] Lee J.W., Kim Y., Han D.H. (2022). LDA-based topic modeling for COVID-19-related sports research trends. Front. Psychol..

[86] Malinauskas R., Malinauskiene V. (2023). Characteristics of Stress and Burnout among Lithuanian University Coaches: A Pre-Pandemic Coronavirus and Post-Pandemic Period Comparison. Healthcare.

[87] McMorris T., Colenso S. (1996). Anticipation of Professional Soccer Goalkeepers When Facing Right-and Left-Footed Penalty Kicks. Percept. Mot. Ski..

[88] Comas G.N., Bardavio J.S., Nuviala R., Miguel D.F. (2018). Design of an observational instrument for the assessment of the penalty kicks in football and analysis of the obtained results. Rev. Psicol. Deporte..

[89] Lopes J.E., Jacobs D.M., Travieso D., Araújo D. (2014). Predicting the lateral direction of deceptive and non-deceptive penalty kicks in football from the kinematics of the kicker. Hum. Mov. Sci..

[90] Pereira M.R., Patching G.R. (2021). Goal Side Selection of Penalty Shots in Soccer: A Laboratory Study and Analyses of Men’s World Cup Shoot-Outs. Percept. Mot. Ski..

[91] Ford P., Hodges N.J., Williams A.M. (2007). Examining Action Effects in the Execution of a Skilled Soccer Kick by Using Erroneous Feedback. J. Mot. Behav..

[92] Navia J.A., van der Kamp J., Avilés C., Aceituno J. (2019). Self-Control in Aiming Supports Coping with Psychological Pressure in Soccer Penalty Kicks. Front. Psychol..

[93] Vieira L.F., do Nascimento J.R.A., Vieira J.L.L. (2013). Perfectionism and Group Cohesion among Adult Indoor Soccer Players. Rev. Psicol. Deporte..

[94] Donachie T.C., Hill A.P. (2020). Helping soccer players help themselves: Effectiveness of a psychoeducational book in reducing perfectionism. J. Appl. Sport Psychol..

[95] Zoudji B., Thon B. (2003). Expertise and implicit memory: Differential repetition priming effects on decision making in experienced and inexperienced soccer players. Int. J. Sport Psychol..

[96] Reigal R.E., González-Guirval F., Morillo-Baro J.P., Morales-Sánchez V., de Mier R.J.-R., Hernández-Mendo A. (2019). Effects of a Computerized Training on Attentional Capacity of Young Soccer Players. Front. Psychol..

[97] Fontana F.E., Mazzardo O., Mokgothu C., Furtado O., Gallagher J.D. (2009). Influence of exercise intensity on the decision-making performance of experienced and inexperienced soccer players. J. Sport Exerc. Psychol..

[98] E Horrocks D., McKenna J., E Whitehead A., Taylor P.J., Morley A.M., Lawrence I. (2016). Preparation, structured deliberate practice and decision making in elite level football: The case study of Gary Neville (Manchester United FC and England). Int. J. Sports Sci. Coach..

[99] Musculus L., Bäder J., Sander L., Vogt T. (2021). The Influence of Environmental Constraints in 360° Videos on Decision Making in Soccer. J. Sport Exerc. Psychol..

[100] Vu A., Sorel A., Limballe A., Bideau B., Kulpa R. (2022). Multiple Players Tracking in Virtual Reality: Influence of Soccer Specific Trajectories and Relationship with Gaze Activity. Front. Psychol..

[101] Larkin P., O’connor D., Williams A.M. (2015). Perfectionism and sport-specific engagement in elite youth soccer players. J. Sports Sci..

[102] Jordana A., Ramis Y., Chamorro J.L., Pons J., Borrueco M., De Brandt K., Torregrossa M. (2022). Ready for Failure? Irrational Beliefs, Perfectionism and Mental Health in Male Soccer Academy Players. J. Ration. Cogn. Ther..

[103] Glavaš D., Pandžić M., Domijan D. (2023). The role of working memory capacity in soccer tactical decision making at different levels of expertise. Cogn. Res. Princ. Implic..

[104] Hosp B., Schultz F., Kasneci E., Höner O. (2021). Expertise Classification of Soccer Goalkeepers in Highly Dynamic Decision Tasks: A Deep Learning Approach for Temporal and Spatial Feature Recognition of Fixation Image Patch Sequences. Front. Sports Act. Living.

[105] Henriksen K., Stambulova N., Roessler K.K. (2010). Successful talent development in track and field: Considering the role of environment. Scand. J. Med. Sci. Sports.

[106] Dohme L.-C., Piggott D., Backhouse S., Morgan G. (2019). Psychological Skills and Characteristics Facilitative of Youth Athletes’ Development: A Systematic Review. Sport Psychol..

[107] MacNamara A., Collins D. (2015). Profiling, Exploiting, and Countering Psychological Characteristics in Talent Identification and Development. Sport Psychol..

[108] Freitas S., Dias C., Fonseca A. (2013). Psychological skills training applied to soccer: A systematic review based on research methodol-ogies. Rev. Eur. Stud..

[109] VanYperen N.W. (1995). Interpersonal Stress, Performance Level, and Parental Support: A Longitudinal Study among Highly Skilled Young Soccer Players. Sport Psychol..

[110] Mehrsafar A.H., Zadeh A.M., Sánchez J.C.J., Gazerani P. (2021). Competitive anxiety or Coronavirus anxiety? The psychophysiological responses of professional football players after returning to competition during the COVID-19 pandemic. Psychoneuroendocrinology.

[111] Kroshus E., De Freese J. (2017). Athlete Burnout Prevention Strategies Used by U.S. Collegiate Soccer Coaches. Sport Psychol..

[112] Moll T., Davies G.L. (2021). The effects of coaches’ emotional expressions on players’ performance: Experimental evidence in a football context. Psychol. Sport Exerc..

[113] Çetinkalp Z.K., Turksoy A. (2011). Goal Orientation and Self-Efficacy as Predictors of Male Adolescent Soccer Players’ Motivation to Participate. Soc. Behav. Pers. Int. J..

[114] Zourbanos N., Haznadar A., Papaioannou A., Tzioumakis Y., Krommidas C., Hatzigeorgiadis A. (2015). The Relationships Between Athletes’ Perceptions of Coach-Created Motivational Climate, Self-Talk, and Self-Efficacy in Youth Soccer. J. Appl. Sport Psychol..

[115] Traclet A., Souchon N., Rascle O., Coulomb-Cabagno G., Dosseville F. (2008). Aggressor and Victim Perspective-Related Differences in Perceived Legitimacy of Aggression in Soccer. Percept. Mot. Ski..

[116] Nicholls A.R., Earle K., Earle F., Madigan D.J. (2017). Perceptions of the Coach–Athlete Relationship Predict the Attainment of Mastery Achievement Goals Six Months Later: A Two-Wave Longitudinal Study among F. A. Premier League Academy Soccer Players. Front. Psychol..

[117] Van Yperen N.W., Dankers S., Elbe A.-M., Sanchez X., Otten S. (2020). Perceived inclusion in youth soccer teams: The role of societal status and perceived motivational goal climate. Psychol. Sport Exerc..

[118] Hassan M.F.H., Morgan K. (2015). Effects of a Mastery Intervention Programme on the Motivational Climate and Achievement Goals in Sport Coaching: A Pilot Study. Int. J. Sports Sci. Coach..

[119] Gouttebarge V., Aoki H., Kerkhoffs G. (2015). Symptoms of Common Mental Disorders and Adverse Health Behaviours in Male Professional Soccer Players. J. Hum. Kinet..

[120] Baltzell A., Caraballo N., Chipman K., Hayden L. (2014). A Qualitative Study of the Mindfulness Meditation Training for Sport: Division I Female Soccer Players’ Experience. J. Clin. Sport Psychol..

[121] Gilbourne D., Richardson D. (2005). Tales from the field: Personal reflections on the provision of psychological support in professional soccer. Psychol. Sport Exerc..

[122] Reilly T., Gilbourne D. (2003). Science and football: A review of applied research in the football codes. J. Sports Sci..

[123] Reilly T., Bangsbo J., Franks A. (2000). Anthropometric and physiological predispositions for elite soccer. J. Sports Sci..

[124] Becerra Patiño B.A., Sarria Lozano J.C., Palomino F.J. (2023). Characterization of variables associated with sports performance: Inter-disciplinarity in women’s soccer in Colombia. J. Phys. Educ. Sport.

[125] Rollo I., Carter J.M., Close G.L., Yangüas J., Gomez-Diaz A., Leal D.M., Duda J.L., Holohan D., Erith S.J., Podlog L. (2020). Role of sports psychology and sports nutrition in return to play from musculoskeletal injuries in professional soccer: An interdisciplinary approach. Eur. J. Sport Sci..

[126] Behnke M., Tomczak M., Kaczmarek L.D., Komar M., Gracz J. (2017). The Sport Mental Training Questionnaire: Development and Validation. Curr. Psychol..

[127] Hansen A.A., Perry J.E., Lace J.W., Merz Z.C., Montgomery T.L., Ross M.J. (2019). Development and Validation of a Monitoring Instrument for Sport Psychology Practice: The Sport Psychology Outcomes and Research Tool (SPORT). J. Clin. Sport Psychol..

[128] Freemantle A.W., Stafford L.D., Wagstaff C.R., Akehurst L., van Laar D.L. (2021). Development and validation of an in-competition emotion measure: The Brief In-Competition Emotion (BICE) scale. Psychol. Sport Exerc..

